# Gratitude among advanced cancer patients and their caregivers: The role of early palliative care

**DOI:** 10.3389/fonc.2022.991250

**Published:** 2022-10-24

**Authors:** Eleonora Borelli, Sarah Bigi, Leonardo Potenza, Fabio Gilioli, Fabrizio Artioli, Giampiero Porzio, Carlo Adolfo Porro, Fabio Efficace, Eduardo Bruera, Mario Luppi, Elena Bandieri

**Affiliations:** ^1^ Department of Medical and Surgical Sciences, University of Modena and Reggio Emilia, Modena, Italy; ^2^ Department of Linguistic Sciences and Foreign Literatures, Catholic University of the Sacred Heart, Milan, Italy; ^3^ Hematology Unit and Chair, Azienda Ospedaliera Universitaria di Modena, Modena, Italy; ^4^ Department of Internal Medicine and Rehabilitation, Unitá Sanitaria Locale (USL), Modena, Italy; ^5^ Oncology and Palliative Care Units, Civil Hospital Carpi, Unitá Sanitaria Locale (USL), Carpi, Italy; ^6^ Tuscany Tumor Association, Florence, Italy; ^7^ Department of Biomedical, Metabolic and Neural Sciences, University of Modena and Reggio Emilia, Modena, Italy; ^8^ Center for Neuroscience and Neurotechnology, University of Modena and Reggio Emilia, Modena, Italy; ^9^ Health Outcomes Research Unit, Italian Group for Adult Hematologic Diseases (GIMEMA), Rome, Italy; ^10^ Palliative Care and Rehabilitation Medicine, UT MD Anderson Cancer Center, Houston, TX, United States

**Keywords:** early palliative care, cancer, patients, caregivers, qualitative research, gratitude, spirituality, communication

## Abstract

**Objective:**

A cancer diagnosis represents a unique trauma, given its life-threatening, multidimensional, and uncertain nature. Gratitude is a construct representing the emotional state that arises when individuals recognize that a benefit has been received as a result of someone else’s action or a spiritual entity’s intervention. Based on the positive psychological wellbeing, gratitude has been associated with improved health outcomes even in the disease setting. Thus, the models of care that foster gratitude should be adopted in the clinical context. This study aims to explore whether and how gratitude may originate in patients with advanced cancer and their caregivers undergoing early palliative care (EPC).

**Methods:**

We analyzed 251 reports from 133 patients and 118 caregivers describing their clinical experience in two EPC units. The sources of gratitude were identified and ranked based on their frequencies. Words expressing gratitude and words referring to communication and spirituality were collected by means of the Linguistic Inquiry and Word Count software and correlated.

**Results:**

In total, 123 (92.5%) of 133 patients’ and 97 (82.2%) of 118 caregivers’ reports, respectively, included explicit or implicit expressions of gratitude. Gratitude was associated specifically with successful physical symptom management, emotional support, improved attitude toward death, better information, humanity, and the familiar environment. The use of words of gratitude in patients’ reports was positively correlated with the use of words referring to communication (r = .215, p = .026) and spirituality (r = .612, p <.001).

**Conclusion:**

Our results suggest that interventions within the EPC model based on doctor–patient–caregiver communication may allow patients and caregivers to experience a feeling of gratitude, and this may represent a resource to be exploited to improve their physical and psychosocial wellbeing.

## Introduction

A cancer diagnosis and its treatment represent a unique trauma for most patients. The detection of an abnormality on self- or routine examination, the following laboratory tests and screening procedures, and the communication of a life-threatening illness are shocking and cause patients to face an escalation of fear and uncertainty that leaves them vulnerable and apparently with no control over events (1). Once on standard oncology care (SOC), patients are overwhelmed by the side effects (2) that may not get fully addressed by oncologists (3) and trigger cascading consequences on their physical and psychosocial wellbeing (2). Their primary caregivers experience similar feelings, exacerbated by the burden of their responsibility (2). Apparently, there are no reasons to feel grateful in such a situation.

In psychology, gratitude is defined as a transient, emotional state arising from a two-stage process: the recognition that a benefit has been received and the acknowledgment that such a benefit is derived from someone else’s action (4–8). When gratitude is experienced more regularly over time, in the form of a more general disposition in noticing and appreciating positive aspects in the world, it is conceptualized as a personality trait more than an emotional state (9). In any case, it is commonly accepted that gratitude occurs in interpersonal contexts (10). From an evolutionary perspective, it has been proposed that gratitude is functional to identify those who have demonstrated responsiveness to their needs and preferences in order to create bonds with them (11). In some cases, the source of recognized benefits or positive aspects in the world may also be attributed to impersonal or non-human sources like God, nature, or the universe, suggesting that gratitude could also be related to the concept of spirituality beyond that of interpersonal relationship (6, 9, 12–14).

Based on the idea of positive psychological wellbeing, a construct representing positive thoughts, emotions, and strategies people use to evaluate their life positively (15), specific positive emotions like gratitude might be potent predictors of improved health outcomes during the periods of chronic stress, including life-limiting illness (10). In this sense, gratitude has received considerable attention in health research over the last two decades, also in relation to the oncological populations (6, 10, 16, 17). There is a strong literature linking gratitude to psychological wellbeing and positive social relationships, and the research linking gratitude to physical health, although more limited, is insightful (18–20). In consideration of the extreme influence that gratitude might have in a cancer population facing a life-threatening diagnosis, clinicians should adopt the models of care that foster it.

Only recently, the role of gratitude has been investigated in the context of palliative care (7, 9, 18, 21–23). The interest raises from the consideration that gratitude has been specifically linked with psychological constructs relevant for palliative care as anxiety (24) and death anxiety (25, 26), depression (12, 27–30), and psychological distress (30). Recently, Centeno and colleagues (31) analyzed the content of 110 thank-you letters sent from bereaved caregivers to two palliative care units to understand the reasons behind the gratitude felt toward the palliative team. Results showed that caregivers’ gratitude arose from the essential characteristics and principles of palliative care, like humanity, professionalism, emotional support, and holistic interventions, that address the unmet needs usually recognized by patients with advanced cancer and their caregivers (2). The only study on gratitude in palliative care involving patients has been conducted by Althaus and colleagues (9). By administering validated questionnaires to 64 cancer patients on palliative care, the authors found that gratitude arises in this context mainly through relationships with family and friends. They also found that gratitude is positively associated with the health status, quality of life (QoL), and appreciation of life, and a post-traumatic growth dimension and negatively associated with psychological distress, supporting the hypothesis that gratitude may have a positive impact on the QoL and reduce psychological distress in palliative care patients at the end of life.

Early palliative care (EPC) integrates palliative care with SOC early in the course of the disease for patients with cancer and their caregivers (32–34). In this model of care, the interpersonal context, i.e., the relationship between the palliative care team, the patient, and the caregiver, which is expressed by an attentive and honest style of communication, plays a key role (2). High-quality communication is the means by which the palliative care team addresses patients’ and caregivers’ unmet physical, psychosocial, and spiritual needs in the long term, by taking charge early on. It is unlikely for patients with cancer to express spontaneously their doubts and fears, and they are grateful when their physicians are proactive in confronting distressing issues (35, 36). Thus, it could be speculated that EPC could trigger, although unsolicited, an emotional state of gratitude in both the patient and the caregiver towards the palliative team and function as a positive psychological intervention.

In this exploratory study, we analyzed 251 reports from patients with advanced cancer and their caregivers under EPC talking about their clinical experience with the model, in order to verify the hypotheses that an emotional state of gratitude might be commonly encountered among cancer patients and their caregivers on EPC and that the long-term, high-quality relationship and communication with the palliative team as well as the inclusion of spiritual needs among the goals of care may have a role in its elicitation. Specifically, the objectives were to (1) assess the proportion of patients and caregivers feeling gratitude in the EPC context; (2) record their sources of gratitude; (3) identify associations between gratitude and doctor–patient–caregiver communication, as a measure of their relationship; and (4) identify associations between gratitude and spirituality.

## Materials and methods

### Participants

This study was conducted in two EPC units: the outpatient Oncology and Palliative Care Unit, Civil Hospital Carpi, USL, Modena (Italy) and the outpatient Palliative Care Unit, Section of Hematology, Azienda Ospedaliera Universitaria di Modena (Italy). A total of 133 patients with advanced cancer and 118 caregivers of alive or deceased patients were enrolled between July 2020 and June 2022. Patient and caregiver eligibility required at least four visits at the EPC unit, willingness to complete the task, and age ≥18 years. At the time of the enrollment, patients had a life expectancy of more than 6 months and were not on interim evaluations to be referred to hospice or home care. All participants provided written informed consent prior to data collection.

The study was performed in accordance with the ethical standards of the 2013 Declaration of Helsinki and was approved by the Ethics Committee of Modena (N. 0026448/20).

### Study setting

Our outpatient EPC oncology and hematology units were established in 2006 and 2012, respectively, and integrate primary oncology and hematology specialists with a palliative/supportive care team composed of one physician assistant, one fellow, and one nurse specialized in palliative care (PC), to provide comprehensive symptom management and psychosocial, spiritual, and emotional support to patients with cancer and their families, from the time of diagnosis to advanced/metastatic disease according to established guidelines (3, 32, 37, 38). Patients with an advanced/metastatic cancer diagnosis (with distant metastases, in the case of solid tumors, late-stage disease, and/or a prognosis of 6–24 months) with high symptom burden are electively referred by the oncologists to receive an EPC intervention. The EPC team follows on average 20–30 patients/week and each patient on a regular basis one-to-two times/week. Outpatient EPC interventions are integrated with both specialized nurse home care services and hospices (32, 37, 39).

### Procedure

The task was described to patients and caregivers by the EPC team during a dedicated, face-to-face encounter, also to offer easier opportunities to ask for clarifications. Patients completed a self-administered pen-and-paper questionnaire (**Table 1**) in which they were asked to answer three questions about their experience with the disease prior to and during the EPC intervention and possible changes in the perception and expectations of their future, including at the end of life. A fourth, open question allowed them to openly express their thoughts on the topic. The questionnaire was completed once, at a time of their preference and availability during the weekly appointments at the units. They were free to complete it all at once or in separate sessions. Caregivers completed the same task at home. Both patients and caregivers were asked to submit their responses within 1 month. Self-administered questionnaires were chosen over face-to-face interviews as the best option to respond with comfort to possibly painful questions and to anonymize the process, in order to minimize the risk of social desirability and obsequiousness biases. The sample characteristics were collected with the support of our database and chart reviews.

**Table 1:** Questionnaires for patients and caregivers.

Patients
Talk about your disease experience prior to the EPC.
Talk about your disease experience during the EPC.
Talk about your perception and expectations of the future and your thoughts and feelings about the end of life.
Is there anything more that you would like to say?
Caregivers
For how long did your relative come to the EPC Unit?
What do you think EPC treatments meant for your loved one? And what did they mean to you?
Is there an episode you would like to share with us from the period when you were caring for your loved one?
Would you like to add something else?

### Statistical analysis

Descriptive statistics was performed on the sample characteristics.

The analyses were performed separately for patients and caregivers. The answers to the questionnaire of each participant were analyzed together as a unique report. Two researchers, a physician and psycholinguist (EBa and EBo), independently read the reports and analyzed them based on a common strategy involving a three-step process. The first step consisted of categorizing reports as reporting the expressions of explicit gratitude, implicit gratitude, or no gratitude. Reports reporting the expressions of explicit gratitude were considered those in which the respondent wrote words or expressions of gratitude (e.g., “thank you” and “I am grateful for”). Reports reporting the expressions of implicit gratitude were considered those not mentioning, explicitly, words of gratitude, but involving the use of positive, high-intensity words, expressions, or metaphors conveying great warmth and enthusiasm when talking about the experience with the EPC (e.g., “EPC unit has been a *lifeline*” and “These doctors are *outstanding*”). Reports that could not be categorized as reporting the expressions of explicit or implicit gratitude were categorized as reports reporting no gratitude. The second step consisted of identifying reasons for gratitude. Reasons for gratitude were considered those aspects of the EPC experience reported in association to explicit or implicit expressions of gratitude. This means that if participants wrote that, once referred to the EPC unit, the pain was resolved, physical symptom management was not considered as associated to gratitude but more as an expected and given-for-granted result. Conversely, if participants wrote that, once referred to the EPC unit, the pain was resolved, thanks to the palliative care team or that the pain that they have been suffering for years was miraculously resolved in a few days, giving them their life back, physical symptom management was considered as associated to gratitude (explicitly or implicitly, respectively). The third step consisted of aggregating reasons for gratitude into broader categories and ranking them based on their frequency. At the end of the three-step process analysis, the two researchers shared the results and refined them through periodic meetings and discussions.

Quantitative analysis was performed on reports reporting the expressions of explicit gratitude through the Italian version of the Linguistic Inquiry and Word Count (LIWC) software (40). This is a psychometrically validated, automated, text-analysis program that measures the percentage of the use of theoretically defined categories of words in either text or speech (41). By uploading *ad hoc* dictionaries of the language of interest, the words of any target text can be filed into one or more categories and subcategories. Categories and subcategories represent dozens of word domains through which LIWC compiles a text. For example, the word “cried” belongs to five word categories/subcategories (overall affect, negative emotions, sadness, verb, and past tense verb); hence, every time the word “cried” is found in the target text, the scores referring to each of these five categories/subcategories will increase (40). Interestingly, LIWC allows to customize *ad hoc* dictionaries by adding the categories/subcategories of interest.

We coded three categories of interest that allowed us to investigate the relationship between the use of words associated to gratitude (e.g., “grateful” and “thank you”), communication (e.g., “to listen” and “to talk”), and spirituality (e.g., “soul” and “redemption”). While the categories related to communication and spirituality were already coded by the LIWC Italian dictionary, we added the category referring to gratitude that coded words like *gratitudine* (“gratitude”), *grata/o* (“grateful”), *grazie* (“thank you”), *ringraziamenti* (“thanks”), and *ringraziare* (“to thank”). Reports including implicit expressions of gratitude were excluded from the analysis because implicit contents cannot be detected by the software.

Through a series of bivariate Pearson correlations, we correlated the coded category of gratitude with the coded categories of communication and spirituality. In accordance with the exploratory approach of our study, we set a significance level at p = .05.

## Results

A total of 133 patients and 118 caregivers took part in the study, for a total of 251 participants. Among participants who were originally approached, 28 patients and 38 caregivers refused to participate because they were feeling uncomfortable or were not interested, resulting in a response rate of 83% for patients and 76% for caregivers. The patients’ mean age was 68.4 years. A total of 118 patients were diagnosed with solid cancer, whereas 15 had a hematologic malignancy. The mean time receiving EPC was 9.8 months. The caregivers’ mean age was 56.7 years, of whom 93 cares/cared for patients with solid cancer and 25 for patients with hematologic cancer. Additional details are reported in **Table 2**.

**Table 2:** Demographic and clinical/caregiving characteristics of the sample (n = 251).

			Patients (n = 133)	Caregivers (n = 118)
Age at enrollment	Years	Mean (sd)	68.4 (11)	56.7 (13.7)
		Range	35–87	20–82
Sex	Female	n (%)	60 (45.1)	77 (65.3)
	Male	n (%)	73 (54.9)	39 (33.1)
Education	Primary school	n (%)	24 (18)	10 (8.5)
	Secondary school	n (%)	40 (30.1)	22 (18.6)
	College	n (%)	54 (40.6)	43 (36.4)
	Graduation’s degree	n (%)	0 (0)	4 (3.4)
	Bachelor’s degree	n (%)	9 (6.8)	32 (27.1)
	Missing data	n (%)	6 (4.5)	7 (5.9)
Ethnicity	Caucasian	n (%)	122 (91.7)	106 (89.8)
	African	n (%)	0 (0)	1 (0.8)
	Arabian	n (%)	3 (2.3)	2 (1.7)
	Indo-European	n (%)	0 (0)	1 (0.8)
	Missing data	n (%)	8 (6)	8 (6.8)
Religion	Catholic	n (%)	92 (69.2)	81 (68.6)
	Muslim	n (%)	3 (2.3)	2 (1.7)
	Evangelic	n (%)	1 (0.8)	1 (0.8)
	Orthodox	n (%)	3 (2.3)	2 (1.7)
	Jehovah’s Witness	n (%)	1 (0.8)	1 (0.8)
	Animist	n (%)	0 (0)	2 (1.7)
	Atheist/Agnostic	n (%)	25 (18.8)	21 (17.8)
	Missing data	n (%)	8 (6)	7 (5.9)
Cancer diagnosis	Solid	n (%)	118 (88.7)	93 (78.8)
	Head, neck, larynx	n (%)	7 (5.9)	–
	Rectum, sigma	n (%)	3 (2.5)	–
	Colon	n (%)	12 (10.2)	–
	Gastric	n (%)	17 (14.4)	–
	Pancreas	n (%)	9 (7.6)	–
	Breast	n (%)	20 (16.9)	–
	Lung	n (%)	19 (16.1)	–
	Genitourinary (kidney, testis, prostate, ovary)	n (%)	24 (20.3)	–
	Skin	n (%)	2 (1.7)	–
	Sarcoma	n (%)	3 (2.5)	–
	Missing data	n (%)	2 (1.7)	–
	Hematologic	n (%)	15 (11.3)	25 (21.2)
Time since first EPC consult	Months	Mean (sd)	9.8 (13.9)	14 (14.7)
		Range	2–96	2–72
KPS score at first EPC consult	0–100	Median (IQR)	60 (50–60)	–
NRS pain score at first EPC consult	0–10	Median (IQR)	7 (6–8)	–
Active CT at first EPC consult		n (%)	52 (72.2)	–
Status of the patient (alive/deceased) at the moment of the caregiver enrollment	Alive	n (%)	–	81 (68.6)
	Deceased	n (%)	–	37 (31.4)
In case of deceased patient, months since death	Months	Mean (sd) Range	–	13.4 (10.1) 1–36
Relationship to patient	Mother/father	n (%)	–	1 (0.8)
	Spouse/partner	n (%)	–	53 (44.9)
	Daughter/son	n (%)	–	51 (43.2)
	Sister/brother	n (%)	–	4 (3.4)
	Other family members	n (%)	–	5 (4.2)
	Missing data	n (%)	–	4 (3.4)

-, no data; CT, chemotherapy; EPC, early palliative care; IQR, interquartile range; KPS, Karnofsky Performance Status; NRS, numerical rating scale.

Of 133 patients’ reports, 123 (92.5%) include explicit or implicit expressions of gratitude. The remaining 10 (7.5%) did not report expressions of gratitude. However, none reported any complaint and all reported positive feedback.

**Figure 1:**
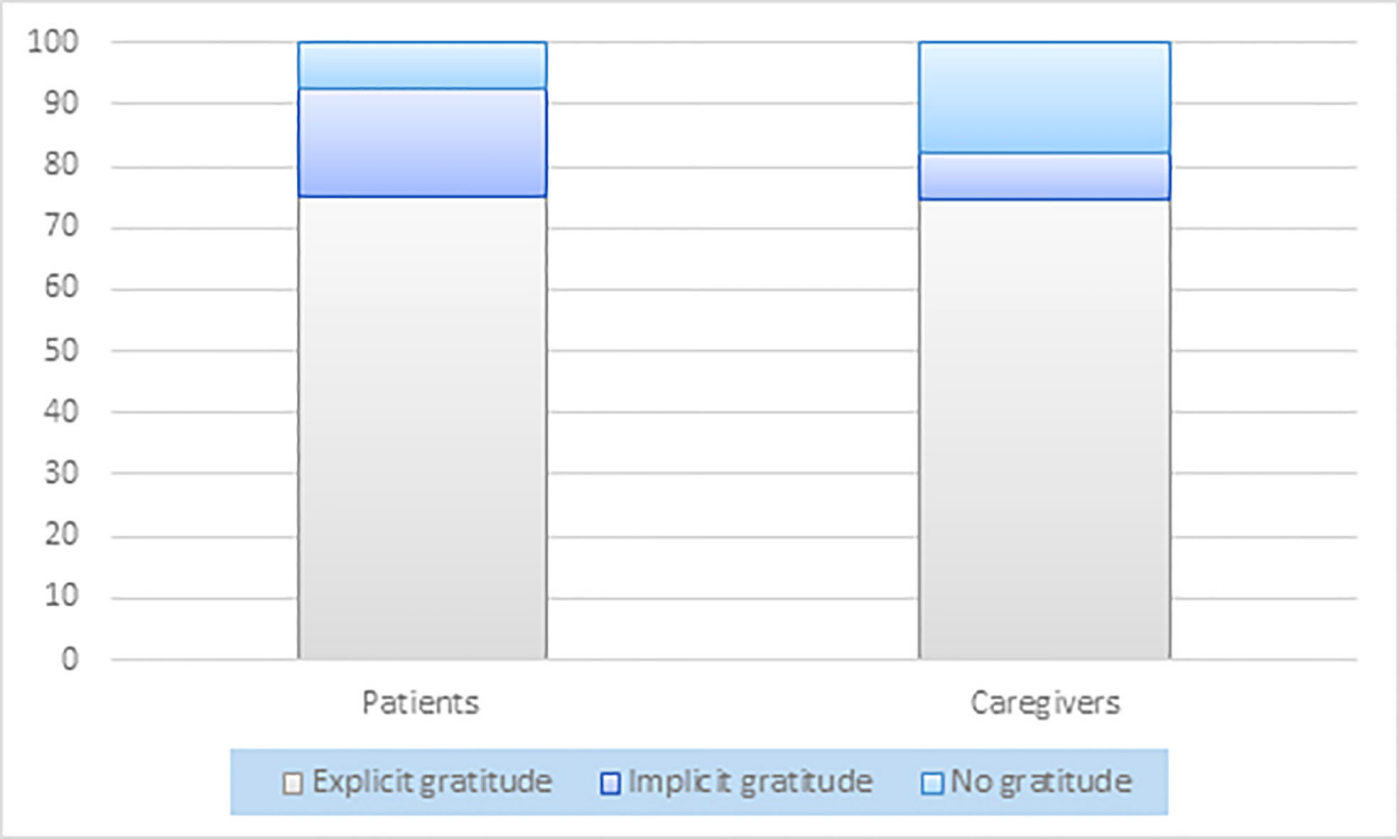
Graphical representation of the percentage proportion between reports showing explicit, implicit, and no gratitude for patients and caregivers.

Expressions of gratitude were explicit in 100 (75.2%) reports and implicit in 23 (17.3%) (**Figure 1** and **Table 3**).

**Table 3:** Quotations of explicit, implicit, and no gratitude from patients and caregivers.

	Patients	Caregivers
Explicit gratitude	001-P-008 “Thanks to doctors’ explanations I was able to go on” 001-P-010 “I thank the team for the emotional support” 002-P-009 “Through the years, my life has improved thanks to the palliative cares” 002-P-022 “I would like to thank from the bottom of my heart for the attention, dedication, care that have always been guaranteed to me, both professionally and humanely. I have always felt entrusted; I would almost dare to say loved.” 002-P-050 “I must say that I am lucky to being able to count on such care, and I must say thank you.”	001-C-001 “A sincere, heartfelt thanks to the people who accompanied us through this very difficult journey. For the humanity, the availability, the professionalism and the patience they have shown day after day.” 001-C-007 “I would like to thank the palliative team for never making us feel alone.” 002-C-017 “I will never stop saying heartfelt thanks for the kindness, respect, humanity as well as the highest professionalism of the clinic staff.” 002-C-009 “As a family member I can only say thank you.” 002-C-016 “Beyond expressing my gratitude, I would like to reiterate the crucial importance of these treatments for everyone.”
Implicit gratitude	002-P-003 “Once arrived here, they gave me my smile back in one week. This is the unit that, as a side effect, has the wellbeing.” 002-P-116 “I must say that here I found a unit that I didn’t even think could exist.” 002-P-102 “The palliative care for me was miraculous, (…). It seemed like a dream to me. I have started to live a life worthy of the name again.” 002-P-006 “To me, the cure received in this unit was truly a godsend”	001-C-005 “All the doctors and the nurses who helped us made us feel like humans and not just the number of a bed, and this really makes the difference.” 002-C-025 “They have been of utmost importance, not only to stem the pain, they have been much more.” 002-C-092 “They have been a lifesafer.”
No gratitude	001-P-002 “In my opinion this clinic was necessary to have a passable standard of living.” 002-P-078 “I did not know the palliative care unit, and I am very sorry, because if I had known earlier I would have been better. I had a great time, pains reduced a lot after only 2 visits. It is an excellent service, I would say necessary.”	001-C-003 “Palliative care was a choice to have some more time, with a good quality of life.” 001-C-011 “Palliative cares have been treatments that allow him to stay active and in a decent physical shape.”

At the beginning of each quotation, the ID of the participant is reported: the first three numbers indicate the unit (001 for the Hematology Unit, Azienda Ospedaliero Universitaria Policlinico, University of Modena and Reggio Emilia Azienda Ospedaliera of Modena and 002 for the Oncology and Palliative Care Unit, Civil Hospital Carpi), the letter indicates patient (P) or caregiver (C), and the last three numbers indicate the recruitment progressive number).

The reasons for gratitude cited by patients can be summarized into six categories: physical symptom management (cited in 83.5% of the reports), emotional support (46.6%), improved attitude toward death (33.8%), better information (24.1%), humanity (22.6%), and familiar environment (12%) (**Tables 4**, **5**).

**Table 4:** Emerging reasons for gratitude and illustrative quotations.

Reasons for gratitude	Percentage	
	Patients	Caregivers
Physical symptoms	83.5%	78%
Emotional support	46.6%	39%
Improved attitude toward death	33.8%	11%
Better information	24.1%	22%
Humanity	22.6%	16.1%
Familiar environment	12%	14.4%

**Table 5:** Emerging reasons for gratitude and illustrative quotations from patients and caregivers.

Reasons for gratitude	Patients	Caregivers
1. Physical symptom management	002-P-009: “When I first came here, I didn’t desire to live because I had so many pains and I was in a severe depressive crisis. Over the years my life has improved thanks to palliative care. As the pain subsided, the wish to live became bigger and bigger.” 002-P-044: “For me they (EPCs) were a salvation, a light. My pains are now more tolerable, my belly has deflated, and I no longer have to do the paracentesis every few days.” 001-P-005: “I am very satisfied with how I have been medicated and informed about the course of the disease. I was brought back to an autonomous and conscious life.”	001-C-007: “We felt so cared for by nurses and doctors who came to our home with an incredible cadence and interacted with us flawlessly.” 002-C-052: “So much relief, my husband had no pain and lived well. And even more, he was peaceful, with me by his side all the time (…). Of course, if he had been suffering I would not have made it.” 002-C-063: “To me they (EPC) represented everything; to see her calm and without pain allowed me to make it.”
2. Emotional support	001-P-004: “I immediately felt welcomed, protected and taken into consideration.” 002-P-003: “This is the clinic which, as a side effect, has the well-being, the feeling well. I come in already knowing that when I will come out, I will be fine. I will be fine in every sense. Physically, emotionally (…).” 002-P-043: “But I would like to say that I feel very well cared for, listened to and understood; in other words, I feel no longer alone but now I am confident. I have full confidence in these people who take care of me (…).”	002-C-092: “I found kindness, hospitality and solidarity, as it should always be when you face such a problem.” 001-C-005: “I start by saying that personally I am not frightened by death, but by the suffering that can be experienced along the way, so this journey reassured me that, feeling helped and “pampered” by doctors and nurses, I will have a lot of relief in dealing with my husband’s disease.” 001-C-006: “I have talked many times with my father about the path we have faced together through palliative care and I can say that for him it was a path of emotional relief, as well as physical, decisive.”
3. Improved attitude toward death	002-P-003: “(…) and life becomes easier, more livable, so when I come here, I don’t think about death.” 002-P-040: “In these last months I have often addressed with the doctor these aspects in conversations, and this is what, personally, I have appreciated the most. Being able to talk about certain things, which is not easy for me (for example with my loved ones), has really helped me a lot to understand and to accept.” 002-P-043: “I am having some interesting conversations with the team on death issues which honestly are helping me a lot in understanding and accepting and getting rid of my fear. I can’t speak to my family about this, thus being able to talk about such topic with those who can actually help you can really be a great help.”	001-C-001: “There was a time when the idea of letting my mother go was unacceptable. (…). It took time, the path of palliative care was also fundamental in this, to learn to let her go and respect her wishes.” 002-C-017: “The beauty of the first meeting with the clinic staff. We went out and, I can’t explain, but we were smiling. Each visit has always been filled with serenity, even when the situation worsened, and the disease progressed. Knowing that you are not alone and that you can count on someone who guarantees you control over your suffering and respect for your will is a lot. And maybe that’s what helped me most in accepting the idea of death.” 002-C-082: “I have been able to prepare myself and it was fundamental for me considering what I will have to face later, at the “end of life”. But I have well understood that, if there hadn’t been this part of early referral here, the so-called “end of life” would have been a real failure. You need time to be able to prepare, otherwise it is all useless.”
4. Better information	002-P-022: “They also allowed me to take the most delicate decisions, for example when I decided to stop oncological therapies because they are no longer effective.” 002-P-027: “These treatments help me to know what I need in order to choose the best care options while respecting my dignity as a person.” 002-P-050: “They talk to me, they listen to me, and they explain things to me, while before no one explained to me what was happening. Now I no longer do cancer treatments because they made me feel bad. Now I’m fine.”	002-C-022: “They helped me in preparing myself for my father’s death, they did it through conversations in which they gradually informed me of the progress of the disease, the prognosis and what to expect. In doing so, they accompanied me and helped me to make the best decision every time, for example the transfer to hospice at the very end.” 002-C-047: “(…), knowing what is happening, knowing the prognosis of the disease, being able to talk to doctors about possible choices, makes me more aware, prepared, and allows me to be able to better follow my mother.” 002-C-051: “If the doctor had not talked to me and informed me step by step of what was going on, I would have gone mad, I would not have made it.”
5. Humanity	002-P-074: “(…) coming here every week and always being able to count on such a trained team, able of making you feel not just a patient, but a person in his entirety (…).” 002-P-093: “I feel respected in my dignity as a human being, before I felt like a number, a tumor, I didn’t feel like a person, as I do now.” 002-P-007: “When I say, “You know, doctor, I feel better with this treatment”, she says “But I WANT you to feel better”, I think that a doctor cannot tell you more than that.”	001-C-005: “All the doctors, the nurses who supported us, made us feel like people and not just the number of a bed and this makes the difference.” 001-C-001: “It was a priority for the doctors to understand what made my mother happy: once she understood that her greatest desire was to go on doing the little things of every day, she was always put in a position to do them and, overall, she was encouraged to do them.” 001-C-001: “I remember that during a visit at which my brother and I were not present, the doctor told my mum some episodes of his personal life referred to his son. When I met my mother in the afternoon, she told me right away and I noticed that the doctor’s confidence had made her happy. It meant that he didn’t just consider her a patient, but a person with whom to joke, with whom to be able to share a private piece of his life.”
6. Familiar environment	002-P-004: “The luck is that a beautiful, almost familiar environment has been created.” 002-P-050: “I love the doctor and the nurse, because they make me feel at home 002-P-045: “By thinking that I always see the kind and smiling faces welcoming me, when I go to the EPC unit, means a lot.”	002-C-053: “My mum feels at home and not in hospital.” 002-C-073: “To see my mother so welcomed, accompanied, as if she was in a family environment, as if she was at home, by very professional but also human doctors and nurses was a surprise. I didn’t know there was such a thing.” 002-C-008: “Dad was a person full of life, sunny and witty, and he was able to pass all this to his fellow adventurers too. Sincere feelings were born with the staff and with the patients, there were moments of important sharing of emotions and feelings.”

At the beginning of each quotation, the ID of the participant is reported: the first three numbers indicate the unit (001 for the Hematology Unit, Azienda Ospedaliero Universitaria Policlinico, University of Modena and Reggio Emilia Azienda Ospedaliera of Modena and 002 for the Oncology and Palliative Care Unit, Civil Hospital Carpi), the letter indicates patient (P) or caregiver (C), and the last three numbers indicate the recruitment progressive number).

Of 118 caregivers’ reports, 97 (82.2%) include explicit or implicit expressions of gratitude. The remaining 21 (17.8%) did not report expressions of gratitude. None reported any complaint, and all reported positive feedback.

Expressions of gratitude were explicit in 88 (74.6%) reports and implicit in 9 (7.6%) (**Figure 1** and **Table 3**).

The reasons for gratitude cited by caregivers were physical symptom management (78%), emotional support (39%), better information (22%), humanity (16.1%), familiar environment (14.4%), and improved attitude toward death (11%) (**Tables 4**, **5**).

Physical symptom management included, on a broader perspective, competence in relieving pain, medical skills, and high levels of scientific competences and professionalism. Emotional support included listening, encouragement, empowering, relieving from the psychological burden, and dedication to participants’ needs. Improved attitude toward death was obtained through discussions, relieving of pain, and positive emotions. Better information referred to prognostic understanding and end-of-life care awareness. Humanity referred to kindness and being treated like persons and not patients. Familiar environment referred to the feeling of calm and peace patients experienced while in the unit.

In patients’ reports, but not in caregivers’ report, the use of words associated to gratitude was positively correlated with words referring to communication (r = .215, p = .026) and spirituality (r = .612, p <.001).

## Discussion

In this study, we investigated the frequency and sources of gratitude as well as their association with communication and spirituality in the context of EPC through the analysis of 251 reports from patients and caregivers, talking about their clinical experience with EPC. To our knowledge, this is the first study to examine the presence of gratitude in the EPC context.

Relative to our first objective, i.e., to assess the proportion of patients and caregivers feeling gratitude in the EPC context, our data show that gratitude arises in most patients and caregivers on EPC. Among patients’ and caregivers’ reports, 92.5% and 82.2% reported explicit or implicit words of gratitude. The study from Centeno and colleagues (31) did not report a proportion between the patients followed by the two palliative care units and the number of caregivers’ thank-you letters received, raising the doubt, as recognized by the authors themselves, that only the most satisfied would write a thank-you letter spontaneously. Our study fills this gap by assessing the frequency of gratitude in the palliative care context not only for caregivers but also for patients. The high percentages of expressions of gratitude found may indicate that gratitude in the EPC context is not linked to personality dispositions but, rather, to an indirect, secondary benefit arising from the well-known, primary benefits attributed to EPC, like symptom control (2, 37, 42), reduced therapy aggressiveness (3, 42–44) and risk of severe pain (32, 37), improved QoL (2, 3, 37, 44–47), mood (2, 3, 37, 44–47), and prognostic awareness (2, 48, 49). Most of SOC interventions have primary benefits on cancer itself but lead to secondary, indirect issues (2, 37). The availability of a model of cancer care that allows, beyond the resolutions of such issues, secondary benefits such as the elicitation of gratitude is of utmost value, given its potential relevance as an indicator of the clinical outcome.

In the oncological setting, patients could still experience positive thoughts and emotions, as shown by studies on post-traumatic growth and benefit finding (50, 51). However, the presence of a positive attitude during the cancer experience often relies on the personal resources of patients and caregivers (e.g., personality and environment). As a positive attitude may be improved by positive psychology interventions (15), these should be systematically provided in the oncology setting. Thus, if the EPC model acts itself as a positive psychological intervention, triggering an emotional state of gratitude, it should be preferred over other models of care.

Relative to our second objective, i.e., to record the sources of gratitude from patients on EPC and their caregivers, we found that gratitude was associated with EPC interventions and specifically with successful physical symptom management, emotional support from the EPC team, improved attitude toward death, better information, humanity, and the familiar environment. Our results confirm and extend the results from Centeno and colleagues (31). Moreover, they mostly overlap with patients’ and caregivers’ perspectives on benefits achieved through EPC (2, 3, 39, 52).

The main EPC interventions soliciting gratitude are symptom management and emotional support for both patients and caregivers. Symptom management often referred to pain relief. The importance of symptom resolution for patients with advanced cancer is in keeping with studies showing that a reduction in pain severity is associated with an improvement in functional status, as early as the first week (37, 42), and that symptom management is necessary to restore physical functioning, mood, and social life (2). Once again, data show that the keystone of EPC is the resolution of physical symptoms. Once symptoms are controlled, more psychological resources are available to cope with the other, equally burdening, issues (2, 53).

Emotional support is the second most cited source of gratitude. Interestingly, emotional support is often reported jointly with better information, i.e., the opportunity to discuss honestly about the disease and its treatments with the medical staff. This may be explained by the fact that emotional support is mainly required when sharing information about the clinical situation. Healthcare professionals often fear removing hope from patients by revealing the truth about their condition (36). However, uncertainty often forces patients and caregivers to take into consideration all the possible scenarios, an extremely energy- and resource-consuming process. Contrary to intuitions, knowing the truth in an emotionally supporting context may help to focus on the real scenario and elaborate it. Moreover, to be aware of the situation allows patients, and even more caregivers, to plan not only the care path but also how to communicate with the other members of the family.

Palliative care was not identified as a source of gratitude by patients in the study by Althaus and colleagues (9). Our additional result may be due to our participants’ longstanding involvement in palliative care. The EPC unit was described by some as a habit, an awaited weekly appointment. An early introduction to palliative care may have led to a better relationship and to a stronger positive impact of its benefits. However, it may also be possible that the different methodologies gave origin to the different results. Indeed, Althaus and colleagues explicitly asked their participants which life domains were identified as a source of gratitude, whereas we identified spontaneously reported sources in the context of a questionnaire focusing on the clinical experience.

Conversely, our results are similar to those by Centeno and colleagues (31), whose methodology was similar to ours (31) but involved the traditional, late, and end-of-life palliative care setting and focused only on caregivers. It is possible that patients more than caregivers could require an early referral to palliative care in order to appreciate its benefits and experience a feeling of gratitude for them.

Relative to our third and fourth objectives, i.e., to identify the associations of gratitude with communication and spirituality, as expected, we found that the more patients were grateful the more they talked about their communication with the palliative team and used words associated to spirituality.

The sources of gratitude we identified are or arise from interventions that distinguish palliative care from SOC. SOC does not always contemplate an honest, empathetic, and truthful communication with patients and caregivers on their unmet needs (3). On the other hand, communication is the main means that the palliative doctor has to understand how to support a patient with advanced cancer in achieving the optimal QoL (54). In fact, communication is upstream to all the sources of gratitude identified. Thus, communication should be further promoted in the EPC setting.

Spirituality refers to the way people find meaning and purpose in the world and how they perceive their connection to self, others, the significant, or sacred (55). Illness can trigger spiritual concerns, both existential and religious (56). Thus, spirituality is properly and comprehensively tackled by palliative care, as a mediating variable affecting care outcomes in terms of the QoL and coping resources (56), as extensively demonstrated (57).

The relationship between gratitude and spirituality is not a new topic in literature. Our results confirm data from studies in both oncological and non-clinical settings reporting that the indexes of spirituality are significantly correlated with the frequency of gratitude feelings (58–61). It may be possible that belonging to an organized religion may contribute to eliciting gratitude by improving social support. The spiritual support received from the religious community may lead to higher wellbeing, eliciting, in turn, feelings of gratitude (6, 13, 62). In our samples, only 18.8% of patients and 17.8% of caregivers did not belong to a religious denomination. However, spirituality can also be described as “an intrinsic private relationship with a divine and spiritual transcendence” (6) and no studies investigate the presence of gratitude in a sample of spiritual, non-religious individuals. Literature suggests a link between spirituality and gratitude and between spirituality and well-being (57, 63). Spirituality can therefore be a resource of strength for patients, and it may play an essential role in the relationship between gratitude and wellbeing, during an experience of illness. Thus, it should be evaluated in the EPC setting.

The present study has several limitations. First, gratitude as a personality trait was not assessed in the sample; thus, we cannot exclude that the high expression of gratitude found among our participants is to be attributed to a widespread predisposition to gratitude and not to an emotional state elicited by the model of care. Second, our analyses do not allow us to draw conclusions on cause–effect relationships. A model representing how gratitude works in the context of EPC should be implemented to run more informative regression analyses. Third, the refusal to participate in the study from 17% of patients and 24% of caregivers might be due to or associated with a lack of gratitude, thus biasing the results. However, 7.5% of patients and 17.8% of caregivers took part in the study even though they did not express any feelings of gratitude. Moreover, the referral bias is still lower than that from the study of Centeno and colleagues (31) since we did not look at letters spontaneously sent by grateful families but rather at responses to a questionnaire on the experience with the disease prior to and during the EPC intervention.

Although unsolicited, gratitude may represent a resource in EPC interventions. Thus, its assessment as well as gratitude-based interventions could be useful in the context of EPC Future directions should be focused on the biological links between gratitude and clinical outcomes in the cancer population (15) in the setting of EPC.

## Data availability statement

The raw data supporting the conclusions of this article will be made available by the authors, without undue reservation.

## Ethics statement

The studies involving human participants were reviewed and approved by Ethics Committee of Modena (N. 0026448/20). The patients/participants provided their written informed consent to participate in this study.

## Author contributions

Conception/design: EBo, SB, LP, FG, FA, GP, CP, FE, EBr, ML, and EBa. Provision of patients: LP, FG, FA, ML, and EBa. Collection and/or assembly of data: EBo, LP, FG, FA, ML, and EBa. Data analysis and interpretation: EBo, SB, LP, FG, FA, GP, CP, FE, EBr, ML, and EBa. Manuscript writing: EBo, SB, LP, FG, FA, GP, CP, FE, EBr, ML, and EBa. Final approval of manuscript: EBo, SB, LP, FG, FA, GP, CP, FE, EBr, ML, and EBa.

## Funding

This work was supported by grants to ML from the “Progetto di Eccellenza Dipartimento MIUR 2017”; the “Charity Dinner Initiative” in memory of Alberto Fontana for Associazione Italiana Lotta alle Leucemie, Linfoma e Mieloma (AIL) — Sezione ‘Luciano Pavarotti’ — Modena ‐ONLUS; and the Fondazione IRIS CERAMICA GROUP.

## Conflict of interest

The authors declare that the research was conducted in the absence of any commercial or financial relationships that could be construed as a potential conflict of interest.

The handling editor declared a past collaboration with the author EBr.

## Publisher’s note

All claims expressed in this article are solely those of the authors and do not necessarily represent those of their affiliated organizations, or those of the publisher, the editors and the reviewers. Any product that may be evaluated in this article, or claim that may be made by its manufacturer, is not guaranteed or endorsed by the publisher.
